# Molybdenum Exposure and Semen Quality: Meeker et al. Respond

**DOI:** 10.1289/ehp.0900922R

**Published:** 2009-09

**Authors:** John D. Meeker, Michael P. Diamond, Julia J. Wirth

**Affiliations:** Department of Environmental, Health Sciences, University of Michigan School of Public Health, Ann Arbor, Michigan, E-mail: meekerj@umich.edu; Department of Obstetrics and Gynecology, Wayne State University, Detroit, Michigan; Department of Epidemiology, Michigan State University, East Lansing, Michigan

We appreciate the comments by Sorahan and Sullivan that serve to reiterate the limitations we discussed in our article reporting relationships between metal concentrations in blood and semen quality among 219 men recruited through two Michigan infertility clinics (Meeker et al. 2008). As highlighted in their letter, these limitations included the possibility of chance findings due to multiple comparisons and the sensitivity of the molybdenum assay used, among others. However, dismissing our analysis as a “fishing expedition” is not appropriate, given the animal evidence for adverse effects on male reproductive function in relation to Mo (Institute of Medicine 2001; Jeter and Davis 1954; Lyubimov et al. 2004; Pandey and Singh 2002; Thomas and Moss 1951; Van Niekerk and Van Niekerk 1989; Vyskocil and Viau 1999; Yamaguchi et al. 2007) and other metals. Because evidence exists for numerous metals to be either harmful or beneficial to male reproduction, and because metals may interact with one another synergistically or antagonistically, we conducted this exploratory study to provide the most comprehensive human data to date on multiple metals and semen quality.

Because our study (Meeker et al. 2008) was the first to assess the relationship between Mo and semen quality in humans, we agree that our findings deserve further consideration and scrutiny through future research. An added impetus for further investigation comes from our recent follow-up analysis in which we reported a strong inverse association between Mo and circulating testosterone in these men, an association that was independent of semen quality (Meeker et al. 2009). Given that testosterone is not strongly predictive of semen quality parameters in this population (correlation coefficients < 0.15) or in other populations (Meeker et al. 2007)—but it does play a vital role in reproduction and other functions in males (e.g., metabolic disorders, bone health, endocrine-related cancers)—our findings suggest that multiple modes of Mo action on male reproduction may be possible and should be investigated.

We considered a number of approaches to model the association between metals in blood and semen quality parameters. In our logistic regression analysis (Meeker et al. 2008), we decided that including only men with all three semen quality parameters above reference values (i.e., “normal”) in the comparison group would be most appropriate, because the use of a heterogeneous comparison group that also includes men with compromised semen quality (i.e., at least one parameter below reference) may erroneously dilute the association between metals and semen quality parameters (Hauser et al. 2006). Sorahan and Sullivan’s assertion that the use of this more homogenous comparison group, which had normal levels of all three parameters, would somehow “overload” the comparison group with high-income nonsmokers in relation to the below-reference groups is unfounded since these factors were considered in the multivariable analyses. Finally, in response to Sorahan and Sullivan’s comment regarding our Table 4 (Meeker et al. 2008), in small- to medium-sized studies it is common practice to use the term “suggestive” instead of “significant” in reporting associations with *p*-values between 0.05 and 0.15. There are many instances where relationships with a *p*-value just above 0.05 should not be completely disregarded, especially (as in this case) if they are consistent with results from other statistical analysis approaches or with previous (e.g., animal) research.

In conclusion, our findings (Meeker et al. 2008) may have large public health implications, but we agree that our study has several limitations. Our results need to be replicated in other large human studies, and potential biological mechanisms for the observed associations, and for metal–metal interactions, should be investigated in more detailed molecular epidemiologic, animal, and *in vitro* studies. Research is also needed on important sources, pathways, and routes that result in elevated Mo exposure, whether it stems from ingestion of naturally occurring or industrially related contamination of food and water, intentional ingestion of Mo-containing multi vitamin/multi mineral supplements, potential multipathway exposure resulting from the use of Mo in flame retardants in building materials or consumer products, or a combination of these and other scenarios. Research comparing the utility of measuring Mo in blood, urine, and/or other matrices as biomarkers of Mo exposure over relevant time periods of interest is also needed.
